# Cocoon microstructures through the lens of topological persistence

**DOI:** 10.1098/rsif.2024.0218

**Published:** 2024-11-13

**Authors:** Vira Raichenko, Nikolai Rosenthal, Michaela Eder, Myfanwy E. Evans

**Affiliations:** ^1^ University of Potsdam, Institute for Mathematics, Potsdam 14476, Germany; ^2^ Department of Biomaterials, Max-Planck Institute of Colloids and Interfaces, Potsdam 14476, Germany

**Keywords:** persistent homology, topological data analysis, porous/fibrous/entangled material, microstructure analysis, silk fibre, *Bombyx mori*

## Abstract

Biological materials display a wide array of functionality, often dictated by complicated microstructures. New geometric and topological strategies allow one to describe the microstructures in a precise and systematic way. This article describes the application of topological persistence and other geometric methods to the microstructural analysis of three-dimensional X-ray micro-computed tomography scans of the *Bombyx mori* silkworm cocoons. These methods allow conclusions to be drawn about pore space gradients, silk fibre thickness gradients and fibre alignment within the cocoon. The study demonstrates the applicability of these topological and geometric methods to quantify and characterize fibrous materials.

## 1. Introduction

Silk has been used by humans for several thousand years, e.g. as a medical suture material, for garments, charms and musical instruments [1–5]. While silk is produced by many species of insects and spiders, the silk used by humans for textiles originates from caterpillars of silk moths. More than 500 silk moth species among the Lepidoptera (butterflies and moths) are known, but only a few are used for commercial silk production, with *Bombyx mori* being the main ‘producer’ [6,7]. The silkworm spins the cocoon out of silk as a casing for its pupal stage in which it undergoes metamorphosis from caterpillar to moth. The shape and colour of cocoons are highly diverse and depend on the species and environmental conditions [8]. *Bombyx mori* caterpillars spin the entire cocoon out of a single silk filament (bave), which is up to 1500 m long, spinning from the outside to the inside creating a multi-layered structure [9]. The silk bave is a protein-fibre composite consisting of two fibroin filaments (brin). These are embedded in sericin, acting as the matrix/glue holding the spun filament network in place [10].

Research into the cocoon’s function shows possible oxygen, carbon dioxide and water vapour gating properties, as well as potential insulation against heat or cold [11,12]. Structural analysis of the *B. mori* cocoon membrane often shows a gradient with decreasing pore size from the outside to the inside, where a decrease in filament diameter is also seen [8]. Fibre dimensions and orientation are fundamental parameters for fibrous structures. They impact the mechanical properties of the structure and characteristics such as the porosity [13–16]. The architecture of the pore space is important for functional properties like thermal insulation or gas exchange [12,17,18] and the gradient in pore size is linked to tear resistance [19]. The fibre orientation in a given layer of the cocoon membrane is described as being uniformly distributed in all directions; it is considered a ‘stochastic fibrous material’ with isotropic mechanical properties [20].

Recent research using micro-computed tomography (µCT) to analyse the cocoon of *B. mori*, however, indicates a possible preferred fibre orientation along the short axis of the cocoon [14]. Until now, all microstructural analyses have been performed on two-dimensional images of either manually peeled cocoon layers or two-dimensional µCT slices [14,21]. To gain a robust and accurate picture of the cocoon’s microstructure, analysis of the three-dimensional orientation of the fibres is necessary. However, three-dimensional analysis of the orientation of highly interconnected, curved spun bond fibre networks is a challenging task.

The disordered, entangled and porous structure of the cocoons naturally lends itself to a study from a geometric and topological perspective. Topological data analysis offers methods to analyse complex data using theoretical foundations from both topology and data science. The central mathematical concept of topological data analysis is the notion of persistent homology, which gives a robust characterization of connected components, loops and cavities present in data.

The concept of persistent homology arises concurrently in the separate works of Frosini [22], Robins [23] and Edelsbrunner *et al.* [24] in the 1990s. It provides a characterization of shape and topology of a structure using robust mathematics, and when paired with statistics, it becomes a central idea in topological data analysis. The stability of persistent homology makes its application robust with respect to noise, deformations and interpretability of the results. It allows us to extract the features of the data that are the most prominent, or those that are *persistent*, ignoring irrelevant noise and fluctuations. Persistent homology has been previously used to describe porous materials when the pore space is a complicated network-like shape [25–27], further enabling the study of physical processes in disordered materials [28,29]. Topological data analysis has also been used to predict the physical properties of the effective elastic modulus of porous media in rocks [30,31].

**Figure 1.**
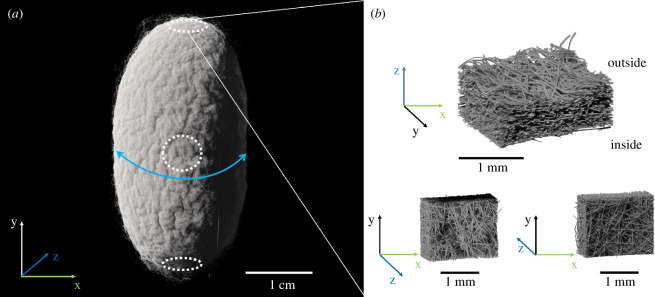
(*a*) A colorized rendered image of the X-ray μCT scan of a cocoon of *B. mori*. The blue arrow indicates the short axis or equator of the cocoon. White circles mark the position of samples cut from the two poles (north at the top, south at the bottom) and the equator of the cocoon. (*b*) Rendered greyscale images of the µCT scans of the marked sample at 2 µm voxel size.

In our case, persistent homology enables us to robustly characterize topological and geometric features of the pore space and fibres of the cocoon, which is what we do in this paper for µCT scans of *B. mori* cocoons (figure 1). We demonstrate the use of persistent homology for extracting geometric information about the pore space and estimating the micro-structural characteristics of the fibres. Such an approach reveals a gradient in the pore sizes through the thickness of the cocoon. We show the gradient in fibre size. Finally, we demonstrate in a robust way that the *B. mori* cocoon is built as a layered structure with a uniform distribution of fibre orientations.

## 2. Topological persistence

Homology is a mathematical idea that describes shapes by counting the number of connected components and holes in different dimensions. For example, the homology of a sphere and a torus are not equal, which tells us that these two shapes are fundamentally different; the torus has a hole through its middle, and the sphere does not. Persistent homology considers this concept in relation to data rather than smooth shapes, identifying topological and geometric patterns that describe the ‘shape’ of the data. This is done by defining a *filtration*, which is a sequence of nested sets related to the data. The persistence of topological features of the sequence of sets through the filtration gives rise to persistence homology. For a binary dataset, a filtration can be constructed using the signed Euclidean distance transform (SEDT), which has been used previously in analysing material microstructures using persistent homology [32,33]. For each voxel in the pore space of the binary dataset, the SEDT returns the distance to the closest voxel on the interface between the object and the pore. For each voxel in the fibre, the SEDT returns the negative value of the distance to the same interface.

**Figure 2.**
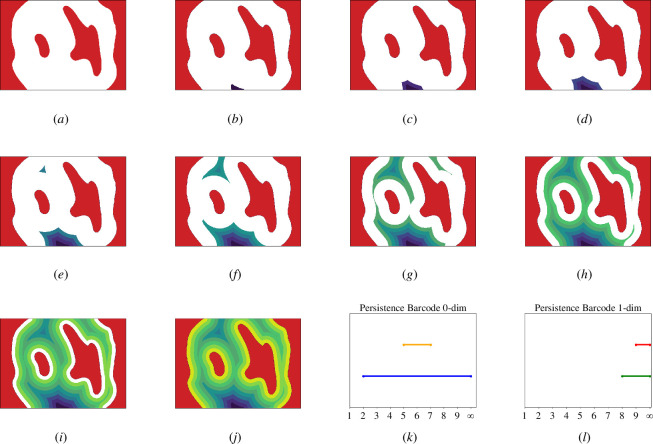
(*a*) A two-dimensional porous pattern, where the white area represents the pore, and the red area is a solid material. Panels (*b*)–(*j*) show level sets of the signed Euclidean distance transform growing from deep in the pore space to the solid red domain. The dark purple region represents the area most distant from the red solid, and the yellow region is the area closest. Each coloured stripe is the area that is equally distant from the solid, where all points have a distance not more than a certain small threshold. (*k*) The zero-dimensional persistence diagram for the filtration shown in images (*b*)-(*j*), represents the persistence of connected components through each subsequent coloured layer. The first blue horizontal line is generated by the first connected component which appears at the second step of the filtration (*b*). The second yellow line corresponds to the connected component that appears at the fifth step of filtration (*e*). These two connected components merge at step seven (*g*), which signifies the ‘death’ of the shorter yellow bar. (*l*) The one-dimensional persistence diagram for the filtration, which describes the persistence of enclosed holes in each subsequent coloured layer. In the eighth step, the level set has one hole, which gives birth to the green bar, whereas the red bar is generated by the second hole which appears in the ninth step.

Figure 2 shows a filtration and the resulting persistence information for a two-dimensional binary image of a pore surrounded by a solid region, shown as white and red respectively in the first image. Figure 2*j* show the filtration (the sequence of nested sets) induced by signed Euclidean distance, growing from deep in the pore space towards the solid red domain. The regions of the pore space that are furthest from the red solid are included first, and subsequent regions are added according to distance from the red domain.

The persistence of topological features is shown in the final two plots of figure 2, representing the persistence of connected components and enclosed holes through the filtration, respectively. Each horizontal line in the plots describes a topological feature, which is *born* at its left endpoint (at the value it first appears in the filtration), and dies at its right endpoint (at the value it disappears in the filtration). We always choose to continue the bar that appeared earlier. Finally, at the end of our filtration, which formally can go to infinity, we are left with one connected component. Each of these horizontal lines can be collected into a plot of birth on the x-axis and death on the y-axis, called a persistence diagram. For three-dimensional images, we have non-trivial persistence diagrams in three dimensions. The significance of each topological feature is measured by the absolute value of the difference between birth and death for that feature, with more persistent features having a larger difference. All non-persistent features are concentrated around the diagonal, and these are characterized as noise or fluctuations. We can simplify the diagrams by deleting non-persistent features up to a certain threshold.

**Figure 3.**
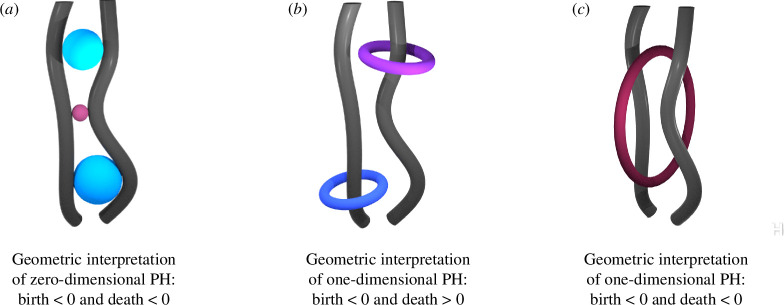
A visualization of the geometrical interpretation of persistence diagrams in different dimensions. Mathematically speaking, each point in a persistence diagram corresponds to a cycle, the notion of a cycle can be geometrically interpreted for specific examples. Here, we demonstrate the intuition behind the structure of cycles in the case of silkworm cocoon data, or more generally fibrous material. Grey wires represent the fibres and the colourful spheres and tori represent the geometrical interpretations of cycles coming from specific regions in persistent. PH; persistent homology.

Persistence diagrams have different geometric interpretations in each dimension. For a three-dimensional dataset, the zero-dimensional diagrams summarize the evolution of connected components, where each birth signifies the appearance of a new connected component in the filtration, and each death tells us when two connected components merge. The one-dimensional diagrams can then be interpreted as the evolution of loops. If a point in the diagram has a negative birth and positive death, it means that this point represents the existence of a loop around the fibre in the level set. When a point has negative birth and negative death, there exists a loop in the pore space that lies in a big pore and dies when fibres get closer to each other. A visualization of these structures is shown in figure 3.

In this article, persistent homology is used on binary three-dimensional data to extract information about connected components and one-dimensional holes.

## 3. Methods

### 3.1. Micro-computed tomography of *Bombyx mori* and resulting data structures

Micro-CT scans were employed for acquiring three-dimensional volumes of dissected cocoon samples. Samples of 4 mm diameter were dissected from the equator and poles of seven cocoons (see figure 1) approximately 5 days after the animals started spinning, using a biopsy punch (KAI Europe GmbH, Solingen, Germany). The resulting silk discs were glued on a carbon stick holder for µCT scanning. The scans were conducted using an EasyTom Nano 160 system (RX solutions, Chavanod, France), equipped with a nanofocus X-ray source Hamamatsu L10711-03 (Hamamatsu Photonics K.K., Iwata, Japan). The scanning parameters were set to a voltage of 55 kV, tube current of 85 µA and voxel sizes of 2 µm and activated anti-ring shift. The frame rate was 1, with an average over eight frames for a total of 1792 projections for each scan. A total of nine scans were obtained, seven for equatorial samples and two for north and south pole samples. The north pole of the cocoon is defined as being at the head side of the pupa, and the south pole is on the abdominal side (see figure 1). The process of reconstruction from projections to tomographic slices was executed using the XAct software in version 22.11 (RX Solutions, Chavanod, France). All reconstructions involved spot correction, geometric x-shift correction and small angle X-ray diffraction correction (phase contrast correction).

Analysis was performed on seven samples from the equator of the cocoon (Samples A–G). Each sample’s inner wall is naturally concave, while the outer surface is convex. To study how the cocoon’s microstructure evolves from the inner to the outer surface, we want to look at the parts of the cocoon that are equidistant from the innermost wall. Therefore, samples A–G were further subdivided to account for the curvature of the cocoon. In the planes XZ and YZ, the image was cut with the step of 600 µm along the axes Z and Y, respectively, which gave us samples of the size 600 × 600 µm × *z*, where z is the thickness of the cocoon. At this scale, the inner wall of the subdivided samples is close to flat. Finally, we rotated each piece so that the inner wall of the cocoon is parallel to the XY plane.

**Figure 4.**
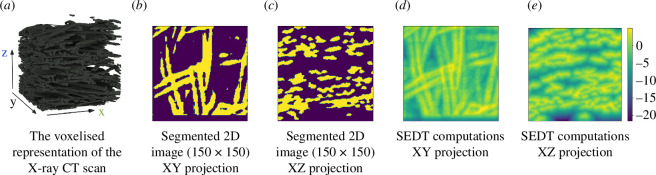
(*a*) A visualization of the voxelised data obtained from the µCT scans, where black voxels in the dataset represent fibres. (*b*)–(*e*) A two-dimensional slice of a segmented image of the silkworm cocoon scan (2 µm) and its SEDT. In the segmented image, the yellow part corresponds to fibres, and the purple one to the background. On the SEDT images the colours are fitted between the negative values of the distance in the pore space and the positive distance in the fibre space and the gradient scale is presented on the colour bar. The axis of the colour bar has the unit of distances between the centre of voxels, e.g. the distance between two neighbouring voxels equals one voxel.

For computing SEDT and persistence diagrams on these samples, we are using the diamorse library [34,35]. This software was previously used to analyse the pore space of the sandstones [30,33,36]. Figure 4 shows a segmented piece of the cocoon alongside two-dimensional slices of the sample along the axis perpendicular to the thickness direction of the cocoon and the respective SEDT calculations.

Further processing steps were required for the fibre analysis. We used a Matlab algorithm to extract a skeleton of the fibre domain of the cocoon, which works best if the sample does not have small holes enclosed in the fibre region. We observed that the pore space was typically fully connected and used this to remove small cavities from the fibres. That the pore space is a fully connected network that percolates the structure is an important property of the cocoon. In our finite sample sizes, the fibre domain is not always fully connected, where separate connected components are typically separated by extended pores. This could be an artefact of our samples being cut to small sizes, where the fibres could reconnect outside of the physical sample domain, making it difficult to draw a strong conclusion here. Using this observation, only those clusters of fibres were extracted that consisted of more than 100 voxels. In this way, we can minimize artefacts in the medial axis. The processing was done using the libraries in Python: NumPy [37], SciPy [38] and connected-components—three-dimensional [39].

**Figure 5.**
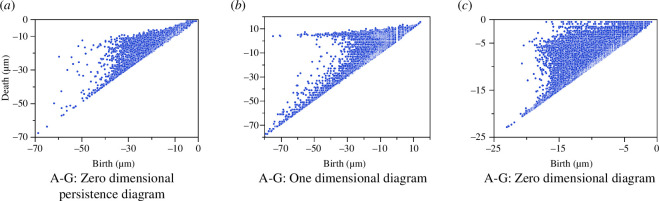
Persistence diagrams of the cocoon Samples A–G in zero and one dimensions. The x-axis is the value of birth and the y-axis is the value of death. The minus sign comes from the negative values of the SEDT, and the absolute values correspond to the physical sizes of the structure and are measured in µm. Panels (*a*) and (*b*) correspond to the case where pore space is a foreground and fibres are the object in the segmented image. Panel (*c*) corresponds to the inverted image where fibres are in the foreground.

We compute persistent diagrams in zero and one dimensions, which are shown in figure 5. Each point in the zero-dimensional diagram can be considered as the maximum included sphere that fits within relatively large pore pockets, where the radius of the sphere is given by the absolute value of the ‘birth’ coordinate (in µm). The one-dimensional diagram summarizes the loops within the pore space through the filtration.

## 4. Results

### 4.1. Pore space analysis

The connectivity of the pore space geometry of the cocoon enables water transport through the channels [17]. It was previously observed that the silkworm cocoon and in particular the *B. mori* cocoon has a gradient in pore size going from the outer to the inner part of the cocoon. Often the layer analysis of the silkworm cocoons requires peeling the layers [40]. Here, we extract the information about the pore space directly from the scans from a piece of cocoon whose inter-layer bondings stay intact.

We start by dividing our samples into layers. The chosen thickness of the layers must be sufficiently large so that we have enough pores within each layer, thus each layer has a thickness of 90 µm (45 voxels). We chose the first five layers to be of the same size starting at the inner wall, and the sixth layer is the remaining material on the outer surface of the cocoon, which has variable thickness due to the non-uniform thickness of the cocoon across the samples.

**Figure 6.**
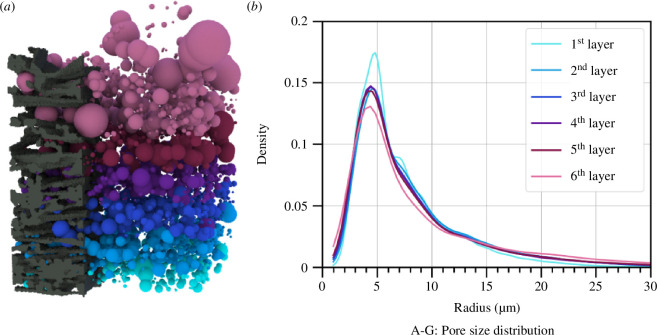
(*a*) The maximal inscribed sphere of each pore of the sample is shown, coloured by layer. The black part shows a small section of the fibres of the sample. (*b*) The kernel density distribution of the pore sizes in different layers of the cocoon, where the pore size radius is shown along the x-axis, coloured as in (*a*). The peaks of the curves are at the value 4.2±0.2 µm, and the height of the peaks decreases from the innermost layer to the outermost.

We analysed the pore space of the cocoon samples using a geometric interpretation of the zero-dimensional persistence diagram (figure 5). Defining distinct pores in a network-like domain is difficult; however, persistent homology can attempt this in a robust way. We can quantify the pore size gradient by observing the distribution of sphere radii within the different layers of the cocoon that we defined earlier. Figure 6 shows each of the pore sizes represented by a sphere, coloured by layers.

The density distribution (kernel density curves) of pore sizes within each layer, averaged across all samples A–G, is shown in the plot in figure 6. The peaks of the distributions are concentrated around the value of radius 4.2±0.2 µm, where the height of the peaks decreases from the innermost layer to the outermost. The standard deviation of the radius within layers ranges between 0.2 and 0.4 µm. The tail height of the distribution increases from the innermost layer to the outermost layer. Since the distribution is skewed, we consider the second moment with standard deviations within layers of each distribution as a measure of the variance of the distribution, which are 18 ± 7 µm (first layer), 30 ± 12 µm (second layer), 33 ± 25 µm (third layer), 35 ± 47 µm (fourth layer), 38 ± 28 µm (fifth layer), and 60 ± 25 µm (sixth layer). Together, this shows that the distribution gets lower and broader (towards larger pore sizes) from the inner layer to the outer layer, indicating an increase in pore sizes, consistent with previous studies [14]. The trends in individual samples are similar, with some expected biological variation. Plots of distributions for individual cocoon samples as well as local behaviour of pore space are given in the electronic supplementary material .

### 4.2. Fibres’ size analysis

**Figure 7.**
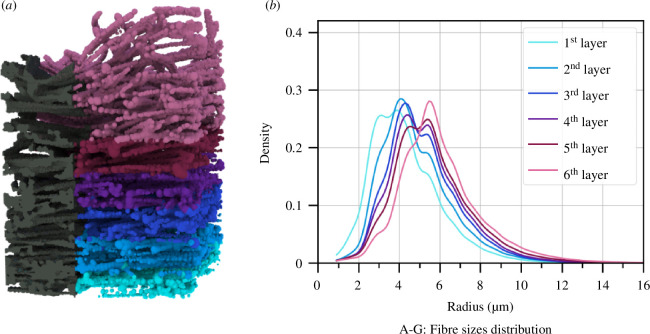
(*a*) The voxelized data on the left shows the fibres coloured by layers. (*b*) The kernel density distribution of the fibre thicknesses in different layers of the cocoon, coloured as in the left-hand image. The corresponding radii for the peaks of the distributions are 3.9 µm (first layer), 4.1µm (second layer), 4.4 µm (third layer), 4.4 µm (fourth layer), 5.4 µm (fifth layer), and 5.5 µm (sixth layer). The minimal fibre thickness decreases from the outer to the inner part of the cocoon.

**Figure 8.**
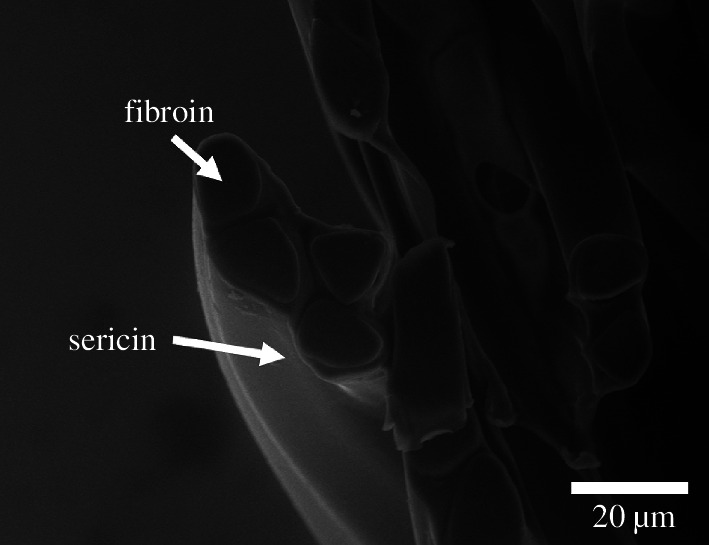
Environmental scanning electron micrograph of the cross-section of the cocoon wall of *B. mori*.

We can estimate the fibre thickness using persistent homology by growing the filtration from the fibre space rather than the pores. The points in the zero-dimensional persistence diagram fill the fibre space with densely packed balls, as shown in figure 7. The radii of these spheres give an estimate of the fibre thickness. Given that the cross-section of the fibre is not circular (see figure 8), the inscribed spheres measure the smallest thickness across the cross-section of the fibre. These results are enhanced by the subdivision of the voxels within the dataset, to improve the sensitivity of the measurement.

The density distribution of fibre thickness within each layer averaged across all samples A–G with corresponding variance, is shown in figure 7. The clearly defined peaks in the distribution of fibre thickness for each layer occur at the following corresponding radii: 3.9 ± 0.6 (first layer), 4.1 ± 0.5 (second layer), 4.4 ± 0.5 (third layer), 4.4 ± 0.7 (fourth layer), 5.4 ± 0.7 (fifth layer), and 5.5 ± 0.5 (sixth layer). The second moments of the fibre distributions are 2.7 µm (first layer), 2.9 µm (second layer), 3.0 µm (third layer), 3.1 µm (fourth layer), 3.1 µm (fifth layer), and 3.7 µm (sixth layer). The fibre thickness has a clear gradient behaviour, similar to that of the pore space, where the minimal fibre thickness increases from the inner layer to the outer layer of the cocoon.

Zhao *et al.* [1] measured fibre thickness for three silk strands from *B. mori* that gave the different ranges for fibre diameter 8.35–22.41, 10.60–22.82 and 9.93–25.74 µm. In a study by Khodarahmi Borujeni *et al.* [15], the fibre diameter was estimated as 21 µm. Another analysis by Chen *et al.* [40] found the fibre thickness to be in the range of 15.01−24.16, 15.35−23.71 and 16.23−24.01 µm. Our estimation is in the lower range compared with these studies, which may be caused by the non-circular fibre cross-section. Compared with the analysis of Pérez *et al.* [41], where they focus on measuring the variability of fibre thickness because of its uneven cross-section, they report the brin diameter ranging from 6.8 up to 16.7 µm with the mean 11 ± 2.1 µm, which is very close to our results. If micrographs are taken in the x or y direction (figure 1) the diameter will be larger, since the two fibroin strands connected by the sericin will be exposed to the objective/camera. The smaller radius of the fibres is only imaged in z-direction. Finally, the preparation of samples for microscopy can affect fibre size measurements by positioning or humidity changes.

### 4.3. Fibre orientation analysis

We use here geometric methods to analyse the orientation of fibres in the cocoon samples. To begin with, the orientation analysis relies on the robust extraction of a skeleton of fibres from the three-dimensional voxelized data. We use *bwskel* function from Matlab that relies on medial axis transform [42]. To increase the reliability of the computation, we compute the medial axis for each image and its three rotations by 90, 180 and 270⁢°. All four medial axes are overlayed, and then we apply the medial axis algorithm to this structure, which gives us a medial axis that reflects the fibres well.

**Figure 9.**
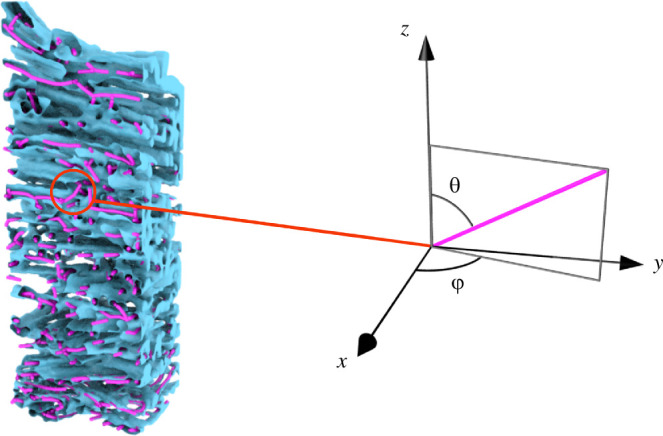
The blue surface is the raw fibre data aligned with the extracted skeleton coloured in pink. On the right, is the definition of polar angle θ and azimuthal angle φ for a piece of the skeleton.

The voxelized skeleton is converted then to a series of vertices and edges, where each vertex corresponds to a voxel centre, and the neighbouring voxels are connected via edges. If more than two edges meet at one vertex, the one with the smaller incidence angle is removed. The vertices and edges are then smoothed using Laplacian smoothing [43], where 10 rounds of smoothing are performed. Additionally, all connected components that are shorter than 20 edges are removed to decrease artefacts from the original image. After this removal, a further 10 rounds of smoothing are repeated. The resulting fibre skeleton is shown in figure 9.

We can measure the orientation of each edge in the skeleton of the cocoon sample using two angles of orientation, namely the angle of deviation from a horizontal plane through the sample, which is the polar angle (θ), and the angle of orientation within that horizontal plane, which is the azimuthal angle (φ). A sketch of this is shown in figure 9.

**Figure 10.**
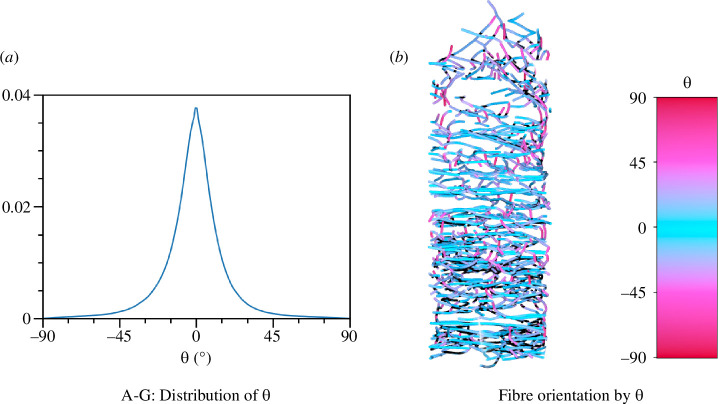
(*a*) The distribution of the polar angle θ over all the layers in *B. mori*. (*b*) An example of skeletonized fibres coloured with respect to θ. The colour bar shows the respective colour coding. One can clearly see the layered nature of the fibres, which hardly deviate from horizontal planes.

A plot of the polar angle for all edges in the skeleton is shown in figure 10*a*. We see that the distribution has a very pronounced peak at zero, which implies that most fibres are oriented within horizontal layers, confirming that the cocoon is a multi-layer structure with very little inter-layer weaving, which can be visually confirmed in figure 10*b*. The large portion of edges with orientation closer to ± 90° comes from the skeletonization of the contact of two fibres lying on top of each other.

With the layered structure confirmed, we can analyse the orientation of fibres within pseudo-layers. This is a process in which it is necessary to have full control of the effects of discretization. If a sequence of vertices and edges is constructed from a voxelized skeleton, the discrete organization of the voxels means that all edges will follow the grid lines of the structure, and the orientation of the edge segments will be in just two directions at right angles to one another. The smoothing process that we apply to the vertices and edges of the skeleton decreases this effect. We first wish to quantify what level of variation in directions we should see in a uniform distribution of fibre directions.

**Figure 11.**
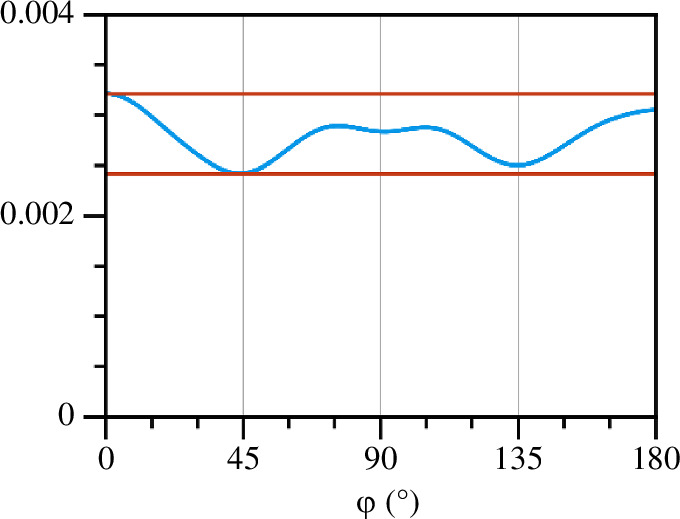
The demonstration of the influence of the computation error for the uniform distribution. Straight lines indicate the maximum and minimum of the discrete error curve.

We estimate this variation by constructing a dataset where lines are uniformly distributed in pseudo-layers. We inflate the lines with a radius of three voxels, which is a typical fibre thickness in our datasets. On this structure, we perform the skeletonization process as described above, and we can now observe how uniform this resulting structure is. An important point here is that we take a circular section of the sample to collate the distribution, which is a necessary choice to avoid biasing the measurement along the diagonals of a square sample (this is discussed further in the conclusion). The distribution of angles within these pseudo-layers is shown in figure 11. For the uniform dataset that we started with, the distribution of angles is a horizontal line across the plot. One can see that the data processing that we perform leads to a small fluctuation around this value, whose magnitude we can use to quantify the variability of the angles due to discretization effects. The magnitude of these fluctuations is also shown on the plot, as the global minimum and global maximum of the fluctuating curve.

**Figure 12.**
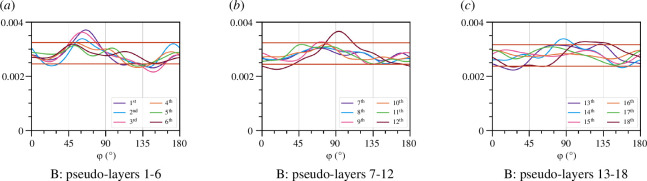
The distribution of φ in the scan B of *B. mori* throughout the pseudo-layers. The pseudo-layers are enumerated from the inner side to the outer side of the cocoon and each pseudo-layers is of the size 40 µm. The horizontal line demonstrates the magnitude of the discretization error coming from the processing and computations. The figure demonstrates the deviation of the fibre distribution from the uniform distribution.

We next look at the angle distribution within thin pseudo-layers of thickness 40 µm of the cocoon samples. Using the skeletonized data, the distribution of edge angles from a chosen direction in the same is computed, and the results are shown in figure 12. The distributions of multiple pseudo-layers are overlayed, and the range of the expected variation for a uniform distribution is shown too (positioned about the average of the distribution curves of the pseudo-layers). From these distributions, we conclude that there is no preferred orientation beyond the expected fluctuations of the distribution curves, and thus that there is no preferred alignment of fibres within the pseudo-layers of the cocoon.

**Figure 13.**
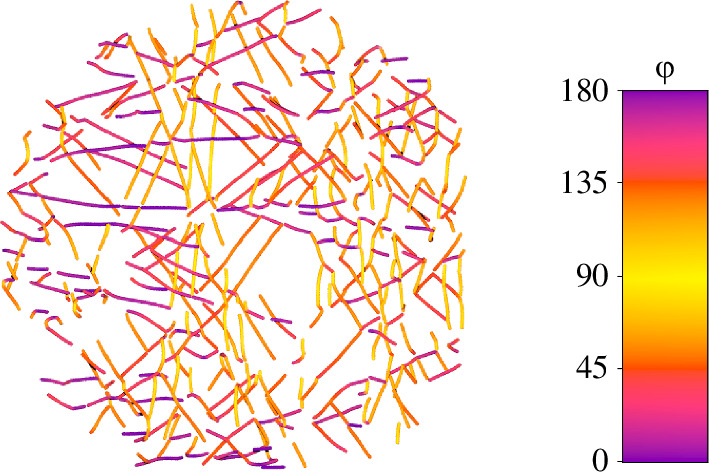
An example of skeletonized fibres coloured with respect to φ. The colour bar shows the respective colour coding.

A visualization of the distribution of fibres within pseudo-layers is shown in figure 13, where the skeleton is coloured by φ.

### 4.4. Extremities of the cocoon

The thickness of the cocoon wall is different at different latitudes. In particular, the wall on the bottom and the top of the cocoon is thinner (see figure 1 for an orientation of the north, south and equatorial parts of the cocoon). Two additional samples were prepared from the north (Sample H) and the south (Sample I) of one of the cocoons (that from which Sample B was also prepared) following the same preparation as for all previous samples. Given the smaller thickness of the sample, we can only split the cocoon into three different layers, the first two layers are 90 µm each, and the last third layer contains the rest of the cocoon structure.

**Figure 14.**
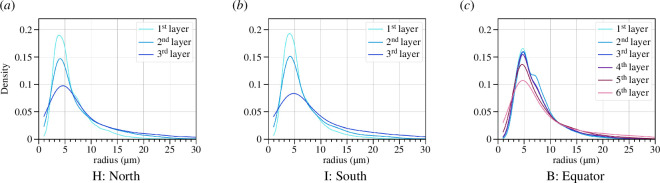
Pore distribution in the same sample of the cocoon in different places for Sample B. (*a*) The distribution of pore size in the north. (*b*) The distribution of the pore size in the south. (*c*) The distribution of the pore size at the equator.

We can observe a clear gradient in pore sizes in both the bottom and top samples, see figure 14. This confirms the idea that the north and south poles of the cocoon display the same pore-size gradients as the equatorial section. We see a difference in the position of the peak though, where the peak is at 4.3 µm for both south and north, while for the sample from the equator, it is at 4.8 µm, which tells us the most common pore size at the extremities is smaller than those at the equator.

**Figure 15.**
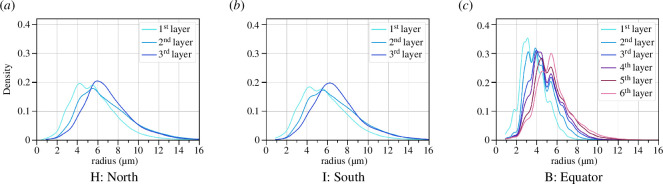
Fibres’ size distribution in the same sample of the cocoon in different places for Sample B. (*a*) The distribution of fibres’ size in the north. (*b*) The distribution of the fibres’ size in the south. (*c*) The distribution of the fibres' size at the equator.

We can also consider the fibre thickness throughout the cocoon sections at the north and south poles of the cocoon, see figure 15. For the sample from the north pole of the cocoon, the fibres’ thickness distribution has its peak at 4.24, 5.41 and 5.71 µm, and for the south 4.27, 4.43 and 5.67 µm, which is slightly larger on average than those seen at the equator.

## 5. Conclusions

In this article, we have demonstrated the gradient behaviour in the pore space in *B. mori* silkworm cocoons through combining methods of three-dimensional microscopy with persistent homology and image processing techniques. We analysed the fibre properties and concluded that the fibre thickness increases from the inner to the outer layer. These results are consistent at the poles of the cocoon as well as around the equator. Finally, we demonstrated through robust techniques that the cocoon is a layered structure and the distribution of fibres cannot be distinguished from the uniform distribution, hence confirming previous results that suggest this. All of these computations were performed on tomography datasets, and no further experimental techniques were required to collect these results. This demonstrates the practicality of using persistent homology and other tools from computational geometry on biological datasets, reducing problems with variability in the experimental conditions.

Measuring fibre thickness is a challenging task due to the soft nature of the fibre as well as its uneven cross-section shape [41]. We demonstrate how to estimate fibre thickness directly from the image data with the advantage of the computational approach, which takes away the environmental variability introduced via sample preparations for the measurements, which has been shown for e.g. sample processing by different persons [16]. Generally, it is advised for CT scans to measure parameters like thickness/diameter in three dimensions, as in two-dimensional slices their apparent values depend on the angle of the cutting plane [44]. In both parts of pore and fibre analysis, the computation involves so-called minimal inscribed spheres and a well-defined analysis through persistent homology, which makes it possible to compare with future analysis for other silkworm cocoons or more generally fibrous materials. We think that the mathematically well-defined techniques of persistent homology give reliable, reproducible measurements of the fibre thickness.

The presented results of this study support the common findings in the literature that the fibre orientation in the layers of the cocoon of *B. mori* is uniformly distributed [40]. This finding is contradictory to a recent analysis of µCT scans, which indicates a preferred fibre orientation towards the short diameter of the cocoon [14]. The conclusion in [14] relied on using normal orientation index (NOI) as a statistical measurement for the preferred orientation. If a distribution has one defined peak, NOI gives a measure of how prominent this peak is. However, in the case of a multiple peak distribution NOI is not a well-defined statistic and it fails to capture the behaviour of a distribution. In addition to this, the shape of the observation window in analysing orientation can play a prominent role. If the image has a square shape, the orientation will be biased towards 45 and 135⁢°: It happens because the lines that have the orientation of 45 and 135⁢° are the longest in the image, and it skews the distribution. Our analysis is performed on circular-shaped images, which removes this bias.

In the computation of persistent homology, one must choose a threshold of what is deemed to be persistent, where everything below the threshold can be considered as noise. Persistence is defined by the absolute value of the difference between the death and birth of a point in a persistence diagram, and the threshold determines a lower cut-off of this value. In our computations, we chose the threshold to be 0.5 for the pore analysis, with our results being robust for small changes in this threshold, and 0 for the fibre analysis, to capture as many measurements for the fine structure.

The results presented in this paper characterize the geometric and topological features of the cocoon on average across many samples, where individual samples show some variation in this average behaviour. Such variance is inevitable in any biological data. The results for each sample individually are presented in electronic supplementary material, for pore sizes, fibre thickness and fibre orientation.

## Data Availability

We have provided data in the electronic supplementary material [45].
